# Refinement of the Lipopolysaccharide-Induced Synovitis Model in Equine Middle Carpal Joints

**DOI:** 10.3390/ani15172474

**Published:** 2025-08-22

**Authors:** Michael J. S. Duggan, Clodagh Kearney, Milda Baltrimaite, Margot C. Labberté, Rory Gibney, Pieter A. J. Brama

**Affiliations:** 1 School of Veterinary Medicine, UCD Veterinary Hospital, University College Dublin, D04 W6F6 Dublin, Ireland; 2 Trinity Centre for Bioengineering, Trinity Biomedical Sciences Institute, Trinity College Dublin, D02 R590 Dublin, Ireland

**Keywords:** equine, lipopolysaccharide, synovitis, model, joint

## Abstract

The aim of this study was to refine the lipopolysaccharide (LPS)-induced synovitis model in normal carpal joints of Thoroughbred horses by comparing two low LPS doses. A further aim was to investigate the relationship between the induced synovitis and lameness. The study design consisted of two phases using nine horses with a unilateral crossover design and a within-animal saline control. Synoviocentesis was performed at post-injection hour (PIH) 0, 8, 24, 72 and 168, allowing for synovial fluid cytology and biomarker analysis. Objective gait and thermographic analysis were used to objectively measure clinical effects. The results demonstrate that injection of either a 0.125 ng or 0.25 ng dose of LPS induces a comparable degree of synovitis in terms of TP, WBC, PGE_2_ and MMP activity at peak values. Statistically significant changes in baseline lameness values were not detected with the 0.125 ng dose, a novel and valuable finding suggesting a comparable degree of synovitis is achieved without significant lameness. All measured parameters had returned to baseline by PIH 168. In conclusion, the findings of this study confirm that this LPS model produces a consistent and reliable synovitis at 0.25 ng and 0.125 ng doses. The reduction in lameness evident at the 0.125 ng dose offers enhanced animal welfare while delivering measurable synovitis. The authors believe that a further reduction in the LPS dose is possible with continued development of a repeated low-dose/slow-release model to better mimic clinical disease.

## Simple Summary

The aim of this study was to improve the lipopolysaccharide-induced model of joint inflammation in the carpal joints of horses by comparing two low doses. A further aim was to look at the relationship between the amount of inflammation caused and lameness. The study was performed in two phases using nine horses. A sample of joint fluid was obtained at five timepoints to enable the analysis. The results show that injection of either a 0.125 ng or 0.25 ng dose of LPS caused a similar amount of inflammation with less lameness in the 0.125 ng dose group. The findings of this study confirm that this model produces a reliable synovitis at both doses. The reduction in lameness at the 0.125 ng dose offers improved animal welfare and the authors believe that a further reduction in the LPS dose is possible.

## 1. Introduction

Intra-articular LPS has been used for many years to experimentally induce lameness in the horse with reports dating back to 1987 [1] and is now a well-established model for the study of inflammatory joint disease and associated therapeutics in the horse [2,3,4,5,6,7,8,9]. The study of osteoarthritis (OA) is challenging due to the variability in presentation and slow progression of the naturally occurring disease process. Hence, the use of animal models has become the mainstay of research methods with a number of models and protocols reported [10]. The horse is considered to be a good model for studying joint disease due to the many similarities to human cartilage in biochemical, biomechanical and structural composition in addition to the biomechanical challenges presented [11,12]. An added advantage of studies utilising equine joint disease models is the high prevalence of OA in equids and the subsequent demand for new and enhanced treatment options.

The dosages of LPS used in the literature vary widely, with many reporting doses in mass concentrations (e.g., μg or ng) which fails to account for potential variations in biological activity of the LPS used which may impact on study results. It is recommended that LPS should be reported in endotoxin units (EU) to account for this, providing a standard for comparison between studies with 1 EU equivalent to 1 IU [13]. Firth et al. report doses ranging from 25 to 45 μg resulting in systemic signs of pyrexia, tachycardia and tachypnoea [1]. This study and the modified protocol (3 μg/joint) by Hawkins et al. reported non-weight bearing lameness which is not representative of the mild lameness typically seen with clinical joint disease [1,14]. Palmar et al. [2] further developed this model by evaluating significantly lower LPS doses (0.125 ng to 5000 ng) in equine middle carpal and tarsocrural joints. LPS doses greater than 0.5 ng resulted in systemic signs including pyrexia and depression with non-weight bearing lameness, whereas those less than 0.5 ng lacked systemic signs and had much milder lameness grades (AAEP) ranging between 1 and 3 [2]. The overwhelming inflammatory response induced at higher dosages does not truly reflect clinical disease and that of subsequent OA, necessitating further work to understand the optimal dose for the study of clinically relevant therapeutics. Firth et al. report white blood cell (WBC) counts of 235 × 10^9^ cells/L with the administration of 25–45 μg of LPS, values that would be more consistent with septic arthritis [1]. There is clear evidence from the literature that a reduction in the LPS dose is associated with a reduction in lameness and systemic response.

The primary objective was to determine the relationship between the LPS dose and the level of intrasynovial inflammatory response. A secondary aim was to investigate the relationship between lameness and the degree of joint inflammation in this model.

We hypothesised that both a 0.25 ng and a 0.125 ng dose of LPS would induce an increase in synovial WBC counts, total protein (TP) and tested biomarkers and that a significant synovitis could be present despite minimal or no clinical lameness. We also hypothesised that this relationship would not be directly proportional, i.e., that halving the LPS dose from 0.25 ng to 0.125 ng would not result in a 50% reduction in synovial biomarker concentrations or lameness but may result in a more clinically relevant degree of synovitis with lower WBC and TP values and minimal lameness, which is more consistent with the presentation of clinical disease.

## 2. Materials and Methods

### 2.1. Study Design

The study design was approved by the animal research ethics committee of University College Dublin and performed under Irish Health Products Regulatory Authority (HPRA) licence (AE18982-P146) at University College Dublin, Lyons Research Farm.

**Figure 1:**
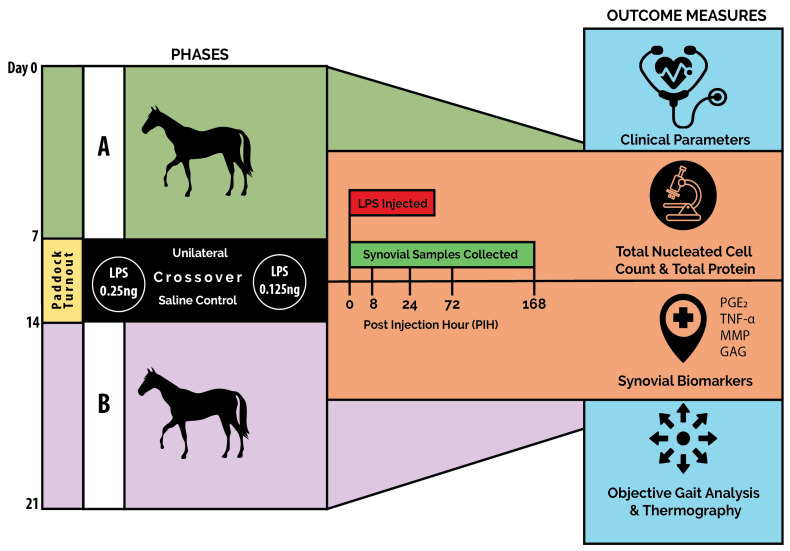
Schematic outline of the 2-phase crossover study design with LPS induction and sampling timepoints indicated. A—1st LPS induction phase, B—2nd LPS induction phase.

The study was conducted in 2 phases (A, B) using a unilateral crossover design, in which each horse received both doses of LPS over the study period. However, only a single dose was used during each phase (unilateral), and only one middle carpal joint (left or right) was injected with LPS at a time, with the contralateral joint serving as the control. In the 2nd phase of the study (B), the LPS was injected into the limb that served as control in phase A. The control used throughout both phases was 0.9% sodium chloride. An overview of the study design is shown in Figure 1.

The same 9 horses were used in both phases of the study, reducing the overall number of experimental animals required. Similarly, each of the 9 horses acted as their own control. A 1 week “washout” period was allowed between each phase, at which time the horses were allowed pasture turnout. Therefore, a 2-week period lapsed between each LPS induction. Previous work by the authors’ group using the same initial 0.25 ng LPS dose showed clinical and synovial inflammatory markers to have returned to baseline within that time frame [15].

All assessment and analysis were performed by the same blinded individual.

### 2.2. Experimental Animals

Nine Thoroughbred racehorses (18 joints) were selected for inclusion in this randomised controlled study. Randomisation was performed using a random number generator. Ages ranged from 6 to 18 years (mean ± SD 13 ± 4 years) with body weights ranging from 475 to 604 kg (mean ± SD 524 ± 44 kg). Eight mares and one gelding were included. Each horse was examined by one ECVS-boarded large animal surgeon (CK), one ECVS large animal surgery resident (MD) and one veterinary intern (MB); their carpal joints were deemed to be within normal limits both clinically and radiographically. Four standard radiographic projections were acquired (lateromedial, dorsopalmar, dorsolateral-palmaromedial oblique and dorsomedial-palmarolateral oblique). During the experimental sampling phases, the horses were kept in stables (4 m × 4 m) on wood shavings. Hay and water were provided ad libitum, and concentrates were fed once daily. During the “off sampling” periods, the horses had access to pasture as a herd and were stabled again the day prior to sampling. The day prior to LPS induction, the horses’ carpal regions were clipped bilaterally, and a reference mark was made at the level of the accessory carpal bone (ACB) using Tipp-Ex^®^ (Société Bic S.A., Paris, France); this mark was maintained for the duration of the study. The Velcro pads for the EquiGait (EquiGait Ltd., Hertford, UK) sensors were applied using double-sided tape and their positions marked using clippers.

### 2.3. Experimental Protocol

#### 2.3.1. Preparation of LPS

The LPS from *Escherichia coli* O55:B5 (L5418; Sigma-Aldrich Ireland Ltd., Arklow, Co. Wicklow, Ireland; Lot: 045M4029V; Potency: 3,000,000 EU/mL) stock solution (3000 EU/mL) was vortexed before being diluted to final concentrations of 0.25 ng/mL (2.5 EU) and 0.125 ng/mL (1.25 EU) in sterile 0.9% sodium chloride less than 60 min prior to the first injection. Vortexing was repeated between each dilution. The final solutions were aliquoted into sterile, clear type I glass vials with rubber self-sealing injection bungs and stored on ice. The LPS solution was inverted vigorously immediately prior to injection.

#### 2.3.2. Induction of Inflammation

Horses were sedated with xylazine hydrochloride (0.2–0.5 mg/kg) intravenously (Chanazine^®^ 10%; Chanelle, Loughrea, Co., Galway, Ireland). The carpal region was aseptically prepared using dilute chlorhexidine and surgical spirit. A 20-gauge 1.5” hypodermic needle was inserted into the middle carpal joint using a dorsolateral approach. The PIH 0 synovial fluid (SF) sample was obtained, and the randomised dose of 1 mL LPS (0.25 ng, 0.125 ng) was injected. The LPS was mixed 3–4 times with synovial fluid within the 1 mL syringe on injection to ensure the whole dose was injected. The same procedure was repeated on the contralateral limb with sterile 0.9% sodium chloride (Aqupharm 1, Animalcare, York, UK) as control.

### 2.4. Clinical Evaluations

#### 2.4.1. Welfare Monitoring

A composite welfare scoring (CWS) system previously developed by our research group was utilised at all of the 12 timepoints (PIH 0, 2, 4, 6, 8, 24, 48, 72, 96, 120, 144, 168) and assigned by one blinded assessor [16].

#### 2.4.2. Clinical Measurements

Carpal joint effusion score (JES), passive carpal flexion score (FS), and carpal circumference (CC) assessment were performed at each of the 12 timepoints by the same person. Middle carpal joint effusion was subjectively graded as previously reported [16,17]. Passive carpal flexion was graded on a 0–3 scale; 0 denoted no reaction, 1 mild pain allowing full flexion, 2 moderate pain intolerant of full flexion and 3 consistently refuses flexion. Carpal circumference was measured using a tape measure (mm) from the same reference mark at the level of the accessory carpal bone. Heart rates, respiratory rates and rectal temperatures were measured as part of the CWS system.

#### 2.4.3. Lameness Evaluation

Objective lameness evaluation was performed at the trot in a straight line on a hard surface using the EquiGait analysis system (Version 4.8, EquiGait Ltd., Hertford, UK) at PIH 0, 2, 4, 6, 8, 24, 48, 72, 96 and 168. Velcro pads were placed on the poll, withers, sacrum, left and right tuber coxae, as per the manufacturer’s instructions. These pads were secured in place with double-sided tape and super glue; their location was marked with clippers to allow accurate replacement should they become dislodged during the study period. Forelimb asymmetry was assessed using poll MnD (minimum difference; mm) [18,19]. A negative value was applied to right forelimb lameness and a positive value to left forelimb lameness to allow for a change from baseline to be calculated and to account for any change in the limb.

#### 2.4.4. Thermography

Thermographic images were acquired using a Variocam HD InfraRed at PIH 0, 2, 4, 6, 8, 24, 48, 72, 96, 120 and 168. The camera was mounted on a tripod set at an angle of 10^o^ and positioned at a fixed distance (1.5 m) from the carpus of each horse in an enclosed area excluding any drafts. The emissivity was set to 1 for all images. The locations of the tripod and feet were marked on the floor to ensure the repeatability of the positioning. The camera was positioned so as to obtain both carpi in one image. Still images were saved at each timepoint. A single circular region of interest was identified in the middle of each carpus to obtain a mean temperature reading (IRBIS 3, InfraTec, Dresden, Germany). The ambient temperature and relative humidity were recorded at each timepoint. To account for variation in environmental temperature changes, the difference between the treated limb and the control limb mean temperatures were calculated as the absolute temperature difference used for statistical analysis.

### 2.5. Synovial Fluid Analysis

Synoviocentesis was performed as outlined above to obtain approximately 3–4 mL of synovial fluid at PIH 0, 8, 24, 72 and 168. The SF was aliquoted into sterile tubes—a plain tube and an ethylenediaminetetraacetic acid (EDTA)-containing tube (1.3 mL). The plain tubes were immediately placed on ice and centrifuged for 15 min at 3500× *g* at 4 °C; the supernatant was aliquoted and stored at −80 °C. A manual white blood cell count was performed on the EDTA sample. Total protein was measured using a refractometer.

#### Synovial Fluid Biomarker Analysis

Commercially available kits were used to measure synovial PGE_2_ (#KGE004B, R&D Systems, Minneapolis, MN, USA) and TNF-α (#ESS0017, Thermo Fisher Scientific, Waltham, MA, USA). In both cases, the synovial fluid was used directly in the respective ELISA sample buffer; for the TNF-α ELISA all samples were diluted twice, while in the PGE_2_ ELISA all PIH 0, 72 and 168, samples were diluted 4 times. PIH 8 and 24 samples were diluted 5 times in order to account for the large concentration range between the expected peak PGE_2_ and the expected amounts pre-inflammation and during recovery. 

Matrix metalloproteinase general activity was measured via cleavage of the fluorescent substrate FS-6 [20]. In brief, 60 µL synovial fluid was added to 240 µL assay buffer (10 mM Tris, 10 mM NaCl, 10 mM CaCl_2_.2H_2_O, with 0.1% PEG-8000 and 0.05% Triton X-100 and adjusted to pH 7.5). A total of 60 µL of diluted synovial fluid was then pipetted into a black 96-well plate in quadruplicate. The amount of 60 µL of the positive FS-6 solution (10 µM FS-6 in ultrapure ddH2O) was then pipetted into the 1st and 3rd wells, and the negative FS-6 (10 µM FS-6 in 10 mM EDTA to inhibit MMP activity) was pipetted into the 2nd and 4th wells in order to have alternating positive and negative wells. The fluorescence was then measured over 12 min with λex at 324 nm and λem at 400 nm, and the slope of the line given by plotting the average positive FS-6 (relative to the negative control) against time was calculated for each sample. This slope in RFU/s was compared between samples as a metric of the MMP activity.

The glycosaminoglycan (GAG) concentration present in synovial fluid was measured via the dimethylmethylene blue (DMMB) colorimetric assay, with an added hyaluronidase digestion step [21]. A total of 20 µL of synovial fluid was mixed with 20 µL of a 100 µg/mL hyaluronidase solution in 50 mM sodium acetate at pH 5.2 and incubated at 37 °C for 30 min. The digested solution was then diluted 10 times in phosphate-buffered EDTA (100 mM Sodium Phosphate Buffer, 5 mM Na2EDTA @ pH 6.5), and 100 µL was then transferred to a 96-well plate in triplicate. An amount of 200 µL Farndale reagent at pH 1.5 was added to the wells and the absorbance read immediately at 530 nm and 590 nm [21,22]. Shark cartilage chondroitin-sulphate was employed as the assay standard.

### 2.6. Statistical Analysis

A sample size calculation was used to estimate the required number of horses based on previous work by this research group and others using similar study designs [4,6,23,24]. The calculation estimated between 7 and 8.07 animals per group were necessary. As a result, 9 horses, including contingency, were used.

A linear mixed model was applied with horse treated as a random effect in each model. Fixed effects included time, treatment and their interaction. Model residuals were examined to ensure they met assumptions of normality and randomness. If the assumptions were violated, appropriate data transformations were performed. Normal distribution was assessed holistically using appropriate numerical descriptive statistics (mean, median and skewness), graphical descriptive statistics (QQ plot, histogram) and statistical tests (Shapiro–Wilk test). Randomness was assessed using a scatterplot of fitted values against residuals, along with a statistical test (Breusch–Pagan test). Tukey’s Honest Significant Difference (HSD) was used for pairwise comparisons. Raw TNF-α data was extremely over-dispersed, with no appropriate data transformation possible. Hence, a between-subjects factor made of all possible combinations to treatment with timepoint was made, applying a Kruskal–Wallis test followed by post hoc analysis controlled for multiple comparisons using Dunn’s test. Due to the ordinal/binary nature of FS and JES data, the relationship was assessed graphically—no formal statistical analysis was performed. A multiple linear regression model was used to assess the relationship between synovial markers and clinical parameters with the initial level set as the reference point. All statistical tests are interpreted using a 5% level of significance (*p* < 0.05). Statistical analysis was performed using R statistical programming language (Version 4.3.2) [25]. No data was excluded from analysis.

## 3. Results

### 3.1. Welfare Monitoring

There were no considerable increases in CWS across any timepoints. Any increases in CWS were mild, with maximum values of 1/16 in four horses, 2/16 in one horse and 3/16 in one horse at one single timepoint. No formal statistical analysis was performed.

### 3.2. Clinical Measurements

No statistically significant difference in carpal circumference (CC) over time between treatments was detected; however, a treatment effect was detected as a main effect only between the 0.125 ng and 0.25 ng (*p* = 0.03) groups. Formal statistical analysis of JES or FS was not performed due to the ordinal nature of the data; however, on graphical assessment there were no differences observed between different LPS dosage groups. The FS peaked at PIH 6 in the 0.25 ng group and at 8 PIH in the 0.125 ng group, with the 0.125 ng dose returning to baseline by PIH 24 and the 0.25 ng dose by PIH 48. Effusion scores peaked at PIH 8 in both groups with a return to baseline at PIH 96. There were no significant increases in HR, RR or rectal temperature across the timepoints when assessed graphically.

### 3.3. Synovial Cytology

**Figure 2:**
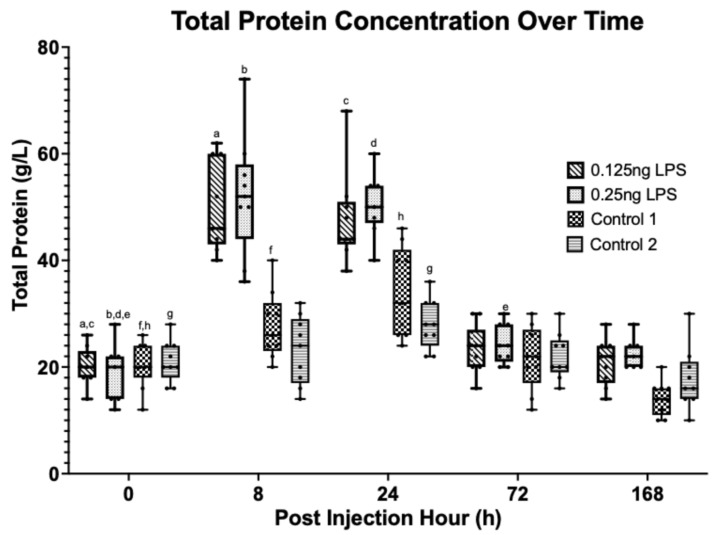
Synovial fluid TP over time between treatment groups. Where statistical significance between treatment groups exists, this is denoted by the respective *p*-value. Lower case letters denote statistically significant differences from baseline, a–f, h (*p* < 0.001), g (*p* = 0.002).

**Figure 3:**
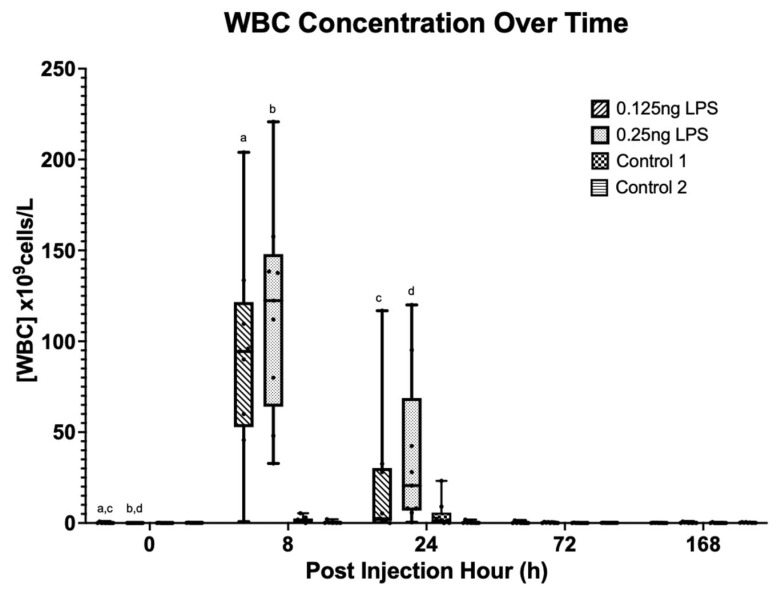
Synovial fluid WBC over time between treatment groups. Where statistical significance between treatment groups exists, this is denoted by the respective *p*-value. Lower case letters denote statistically significant differences from baseline, a–d (*p* < 0.001).

There was a statistically significant increase in the synovial TP [Figure 2] and WBC [Figure 3] concentration from baseline (PIH 0) at PIH 8 and 24 in both the 0.125 ng (*p* < 0.001) and 0.25 ng (*p* < 0.001) treatment groups. No significant difference in TP or WBC was detected between treatment groups. Peak TP and WBC occurred at PIH 8, and by PIH 72 there was no statistically significant difference from baseline TP in the 0.125 ng group and WBC in the 0.125 ng or 0.25 ng group. Total protein concentration in the 0.25 ng group had returned to baseline by PIH 168. The 0.125 ng and 0.25 ng doses of LPS produce a comparable increase in TP and WBC above baseline values at PIH 8 and 24, with both treatment groups returning to baseline values by PIH 168.

### 3.4. Synovial Biomarkers

**Figure 4:**
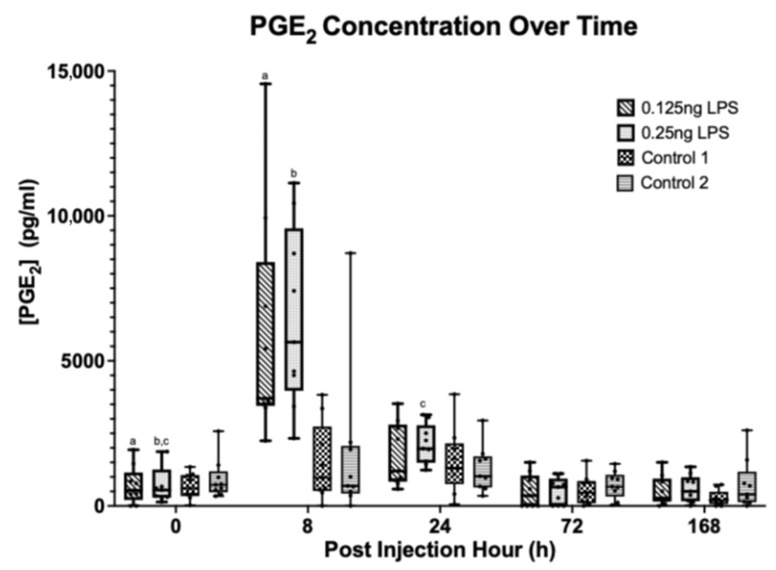
Synovial fluid PGE2 over time between treatment groups. Where statistical significance between treatment groups exists, this is denoted by the respective *p*-value. Lower case letters denote statistically significant differences from baseline, a, b (*p* < 0.001), c (*p* = 0.036).

There was a statistically significant increase in the synovial PGE_2_ [Figure 4] from baseline (PIH 0) at PIH 8 in both the 0.125 ng (*p* < 0.001) and 0.25 ng (*p* < 0.001) treatment groups. There was still a significant increase from baseline at PIH 24 in the 0.25 ng group (*p* = 0.036). Peak PGE_2_ occurred at PIH 8, at which point there was no significant difference in PGE_2_ between treatment groups. At PIH 24 there was no statistically significant difference from baseline PGE_2_ in the 0.125 ng group and at PIH 72 in the 0.25 ng group. The 0.125 ng and 0.25 ng doses of LPS produced a comparable increase in PGE_2_ above baseline values at PIH 8 with the 0.125 ng group returning to baseline values by PIH 24 and the 0.25 ng group by PIH 72.

**Figure 5:**
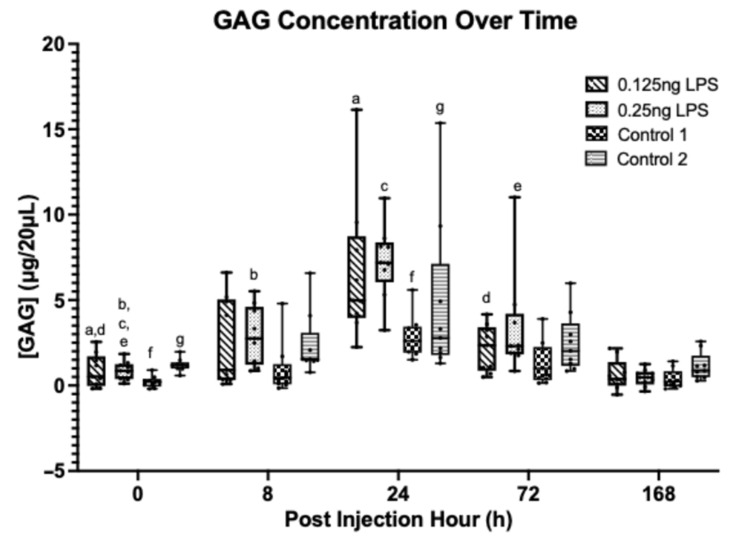
Synovial fluid GAG over time between treatment groups. Where statistical significance between treatment groups exists, this is denoted by the respective *p*-value. Lower case letters denote statistically significant differences from baseline, a–c, e, f (*p* < 0.001), d (*p* = 0.011), g (*p* = 0.002).

A statistically significant increase in synovial GAG [Figure 5] from baseline (PIH 0) occurred at PIH 24 (*p* < 0.001) and PIH 72 in the 0.125 ng (*p* = 0.011) group. However, this increase is not significantly different from the control values at these timepoints. In the 0.25 ng group, a statistically significant increase from baseline (PIH 0) was detected at PIH 8 (*p* = 0.001), PIH 24 (*p* < 0.001) and PIH 72 (*p* < 0.001). However, this increase is only statistically different from control 1 (treatment 3) (*p* = 0.012) and not control 2 (treatment 4) (*p* = 0.064) at these timepoints. Peak GAG concentrations were detected at PIH 24 in both groups; no statistically significant difference was detected between the two treatment groups. The GAG concentrations were no longer significantly different from baseline values by PIH 168. The lack of significance between the 0.25 ng dose and control 2 is likely a result of increased variation in GAG within the control 2 group at the peak timepoint (PIH 24).

A statistically significant increase in synovial TNF-α from baseline was detected for the 0.125 ng (*p* < 0.001) and 0.25 ng (*p* = 0.019) doses of LPS at PIH 8. Values were not significantly different from baseline by PIH 24. There was no difference between the two doses at peak values (*p* = 1). There was a statistically significant difference between the 0.125 ng dose and control 1 (*p* < 0.001) but not control 2 (*p* = 0.647). There was no statistically significant difference between the 0.25 ng dose and either control (*p* = 0.35, *p* = 1).

**Figure 6:**
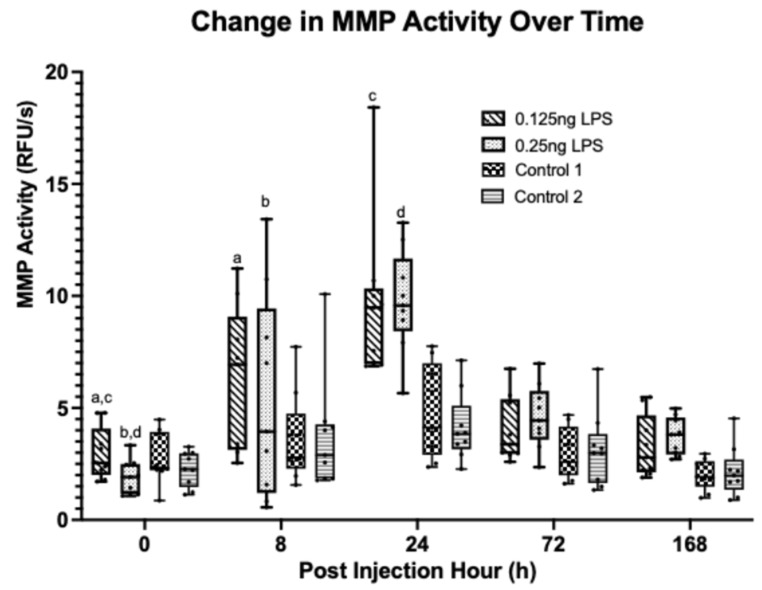
Synovial fluid MMP activity over time between treatment groups. Where statistical significance between treatment groups exists, this is denoted by the respective *p*-value. Lower case letters denote statistically significant differences from baseline, a (*p* = 0.008), b–d (*p* < 0.001).

MMP activity [Figure 6] was statistically significantly increased from baseline at PIH 8 and PIH 24 in both the 0.125 ng (*p* = 0.008, <0.001)) and 0.25 ng doses (*p* < 0.001, *p* < 0.001). There were no statistically significant differences between either LPS dose at PIH 8 (*p* = 0.984) or PIH 24 (*p* = 1.00). At PIH 8 there was no significant difference between either control in the 0.125 ng group (*p* = 0.145, *p* = 0.245) but there was in the 0.25 ng group (*p* = 0.024, *p* = 0.049). However, at PIH 24 statistically significant differences did exist between both LPS doses and all controls (*p* < 0.001). All values had returned to baseline by PIH 72.

An increase in flexion score by one unit was associated with a significant increase in TP and [PGE_2_] by 0.079 g/L (*p* = 0.014) and 5.18 pg (*p* < 0.001), respectively. Increasing joint effusion score by one unit was associated with a significant increase in TP and GAG by 0.089 g/L (*p* < 0.001) and 0.078 ug (*p* = 0.008), respectively. There were no significant associations between WBC or MMP activity.

### 3.5. Objective Gait Analysis

There were no statistically significant differences from baseline lameness at any timepoint in the 0.125 ng group and only at PIH 4 (*p* = 0.002) in the 0.25 ng group, which had returned to baseline by PIH 6. When assessing the data subjectively, nine horses in the 0.25 ng group and seven in the 0.125 ng group recorded MinDiff values of >8 mm.

### 3.6. Thermographic Analysis

**Figure 7:**
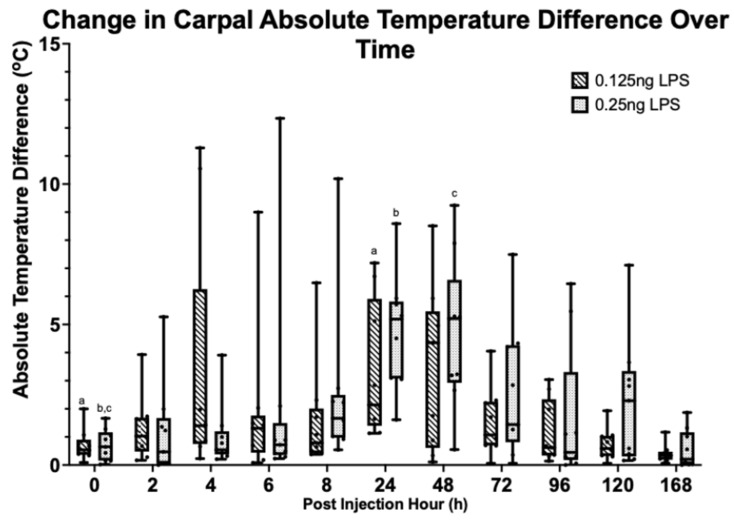
Change in absolute temperature difference (oC) over time between LPS treatment groups. Where statistical significance between treatment groups exists, this is denoted by the respective *p*-value. Lower case letters denote statistically significant differences from baseline, a (*p* = 0.019), b (*p* < 0.001), c (*p* = 0.002).

A statistically significant difference in absolute temperature difference (ATD) at PIH 24 (*p* = 0.019) in the 0.125 ng group and at PIH 24 (*p* = <0.001) and 48 (*p* = 0.002) in the 0.25 ng group was identified. Values had returned to baseline by PIH 48 and 72, respectively. There was no statistically significant difference between treatments [Figure 7].

## 4. Discussion

The findings of this study demonstrate that the injection of either a 0.125 ng or 0.25 ng dose of LPS into the Thoroughbred equine middle carpal joint induces a detectable synovitis with no clear differences between the two doses in terms of TP, WBC or PGE_2_ at peak values. However, the objective lameness measures revealed no significant change from baseline values in the 0.125 ng group. This is a significant finding, suggesting a comparable degree of synovitis is achieved with the lower LPS dose without overt lameness. This finding is particularly novel and valuable given the ongoing quest to refine animal models in order to maximise animal welfare, ideally making models that are subclinical in terms of pain while still representative of disease.

The aim of this study was to assess the effectiveness of lower LPS doses compared to what has been more commonly utilised in the earlier literature [1,2,3,8,14,15,26]. The results clearly show that both the 0.125 ng and 0.25 ng doses of LPS produce a comparable increase in TP and WBC with a peak at PIH 8. The TP had returned to baseline by PIH 72 in the 0.125 ng group and by PIH168 in the 0.25 ng group. The WBC quickly returned to baseline values in both groups by PIH 72. This pattern is also evident in the PGE_2_ values which follow a similar trend, peaking at PIH 8. However, whilst the 0.125 ng group returned to baseline by PIH 24, the 0.25 ng dose exhibited an increase above baseline that persisted through PIH 24 until PIH 72. The trends followed by TP, WBC and PGE_2_ are similar to those reported in other studies using similar LPS doses (0.25 ng to 0.5 ng), where peak values are achieved at PIH 8 [5,8,15].

The MMP activity followed a similar trend to that reported previously by the authors’ group, with a peak activity at PIH 24 [24]. There was no significant difference between the two LPS groups at any timepoint, which again supports that both doses produce a similar degree of synovitis from a biomarker perspective. Analysis of the synovial GAG revealed a peak at PIH 24 in both groups, which is consistent with the MMP activity, but these peaks were no different compared to control values in the 0.125 ng group and only control 1 in the 0.25 ng group. Graphical analysis of control 2 values at PIH 24 revealed considerable variation, which may explain the lack of statistical significance. With repetition or a larger sample size, statistical significance might have been achieved.

An additional objective of this study was to understand the association of lameness with the model using an objective means. The 0.25 ng dose of LPS induced a transient but consistent lameness at PIH 4, whereas the 0.125 ng dose did not induce a significant increase from baseline lameness values during the dynamic assessment at any timepoint when assessed statistically. The fact that seven horses recorded MinDiff values of >8 mm may be misleading, as this does not take into account the baseline status of the horses or any compensatory ipsilateral forelimb lameness that may result from a hindlimb lameness. Hence, in this study, a change from baseline measure was used to eliminate any variation of this kind. Subjectively, there was a marked difference in the degree and consistency of any lameness present in the 0.25 ng group compared with the 0.125 ng group, with the 0.25 ng dose inducing a consistent lameness pattern that was present during the whole dynamic assessment at the respective timepoints. The peak lameness at PIH 4 detected objectively, correlates with the timing of lameness in other studies [8,27,28,29]. When taken together with the synovial cytological and biomarker analysis, synovitis is clearly induced in the 0.125 ng group despite these horses not showing a consistent lameness pattern as a result of the LPS. The PGE_2_ was comparable between groups despite the lack of lameness in the 0.125 ng group. Elevations in PGE_2_ have been associated with lameness located in the fetlock region when compared to sound controls, although there was no association with intra-articular anaesthesia. This suggests that whilst PGE_2_ may be associated with joint pathology, it may not be specific for any one cause [30]. This association with PGE_2_ and joint disease is reported to be independent of WBC count [31]. The findings of this study might suggest otherwise, as WBC counts were comparable for both doses of LPS, although considering the smaller number of samples assessed and the wide individual variation noted, definitive conclusions cannot be drawn.

Some consider the presence of lameness an important outcome measure when studying the efficacy of therapeutics. When the bilateral LPS model is employed, it is not possible to measure lameness. However, a transient peak in lameness is not reflective of clinical disease, which more typically manifests as low-grade inflammation over a period of time, ultimately resulting in varying degrees of lameness [32]. Therefore, the model using a 0.125 ng LPS dose may be more appropriate for the study of prophylactics in early-stage joint disease where lameness is not a consistent feature and where it could be utilised as part of a bilateral model. The development of a low-dose chronic model could be considered using multiple inductions or slow-release preparations of 0.125 ng or lower doses to enhance the clinical relevance of this model [24,33].

The lack of change in the systemic clinical parameters (HR, RR and Temp.) reported in this study is consistent with other studies where low doses of LPS were used [2,34,35].

It has been shown that reducing the dose of LPS results in a reduction in the degree of systemic and intra-articular effects seen [2]. However, this relationship is most likely nonlinear in nature—in this study, halving the LPS dose did not result in halving of the effects. The lower dose of LPS did reduce the degree of lameness exhibited, but many of the other parameters remained unchanged or comparable to the higher dose. It was noted that the higher (0.25 ng) dose of LPS, in the case of TP and PGE_2,_ resulted in a longer time to return to baseline values compared with the lower (0.125 ng) dose (PIH 168 v 72 and PIH 72 v 24, respectively), which may suggest that at these LPS doses some of the effects are evident over a longer duration and not necessarily at greater peak values.

An important factor in this model, and particularly this study design, is the time it takes for the synovial environments to return to baseline when the reuse of the same joint is planned. The results of this study demonstrate that all measured parameters had returned to baseline by PIH 168 if not before, with a further 7-day wash out period assigned. To confirm that all values had returned to baseline, the controls were split by induction, i.e., control 1 for the first LPS induction and control 2 for the second LPS induction; no statistically significant differences were detected at baseline between the controls.

To the authors’ knowledge, this study is the first to include high-level thermographic video analysis of the carpal regions to track the effects of LPS-induced inflammation on skin temperature. Previous studies have used infrared thermometers at much higher doses (3 μg) of LPS, which showed a consistent increase in skin temperatures [29]. Thermography is reported to be ≥10 times more sensitive than palpation in detecting changes in skin temperature [36]. In this study the authors have demonstrated a significant increase in skin temperature at PIH 24 in the 0.125 ng group and at PIH 24 and PIH 48 in the 0.25 ng, with no statistically significant differences between the two groups. Thermography has several limitations, most notably the effects of environmental temperature and heat contamination. To minimise these effects the horses were all assessed in the same shaded stable with no direct sunlight nor significant drafts. Furthermore, thermography was the first measurement performed at each timepoint to avoid any heat contamination. In order to control for changes in environmental temperature and repeated arthrocentesis [37], absolute temperature difference between the treated and control limbs was used. A difference greater than 1 °C likely represents a significant change [38]. The inclusion of thermographic and objective lameness measures is valuable in the refinement of this model by enhancing the objectivity of the outcome measures, particularly around lameness, which is inherently subjective [39]. A further benefit of thermography is that it acts as a non-invasive real-time control, showing the model is functioning as expected when compared with the control limb and adding reassurance ahead of synovial fluid analysis.

The array of synovial biomarkers analysed in this study, whilst greater than in many similar studies, represents only a limited subset of relevant biomarkers and, subsequently, is a limitation. The chosen biomarkers are comparable to other similar studies [5,8,15,34] and were selected to enable comparison. Furthermore, the analysis in this study was limited to five timepoints (PIH 0, 8, 24, 72 and 168), which leaves large intervals to speculation. Where possible, future research should aim to characterise the biomarker profile produced over a greater range of sampling timepoints using this model. This must be balanced between the benefit of increased data and the implications of increased needle sticks on iatrogenic inflammation and horse welfare. It may, therefore, be worth considering different timepoints rather than additional timepoints over the same period. In an effort to maintain consistency in this model, the authors utilised similar timepoints to what has been previously reported [40]. The development of an equine synovial fluid multiplex assay would be advantageous for gaining a greater understanding of the inflammatory processes involved in this model.

There is significant variation in the types of horses used in the LPS literature with mixed breeds [41], Warmbloods [40], Standardbreds [8], Thoroughbreds [42,43] or a combination of Thoroughbreds and Standardbreds [44] all reported. Anecdotal evidence exists amongst practicing veterinary surgeons that some breeds are more stoic (Standardbreds) than others (Thoroughbreds); therefore, the degree of lameness observed may vary, rendering direct comparison of results unreliable. This theory is evident when comparing the lameness observed by Van de Water et al. (2021) [8], who used an LPS dose of 10 EU which is equivalent to 1 ng [45] in Standardbreds, with the lameness observed by [40] using a 0.5 ng dose in Warmbloods. It required twice the LPS dose to achieve the same grade 3 lameness score in the Standardbred population. This is further supported by a 0.5 ng dose of LPS inducing only a “very mild (AAEP grade ≤ 1)” lameness in Standardbreds [46]. In the current study, a 0.25 ng dose of LPS was able to produce an AAEP grade 2 lameness in a strictly Thoroughbred population. The peak (PIH 8) TP and WBC in this study are comparable with those reported for other breeds [Table 1], suggesting the variation in response is likely related to breed-specific pain tolerances, which is consistent with anecdotal reports. However, this cannot be definitively concluded based on the limited data available. Furthermore, the variation in lameness exhibited by some breeds/individuals renders lameness a relatively unreliable outcome measure.

It must be noted that variation in LPS response could also be the result of inter-batch variation, dilution or handling factors and not just related to breed specifics [46], but differences in key synovial biomarkers would be expected. In this study inter-batch variation has been controlled for by using the same LPS batch throughout the experiment. The reporting of dosages stated in mass concentration is not recommended due to the potential for variation in biological activity or potency, so the measure of “endotoxin units (EU)” is more appropriate [13]. Despite this long-standing recommendation, the unit “EU” is infrequently used in the literature. The dosages used in this study are equivalent to 2.5 EU and 1.25 EU, respectively, which represents 25% and 17.5% of the dose used by Van de Water et al. (2021) [8], which supports the belief that some breeds (Thoroughbreds) are more sensitive to the LPS model than others (Standardbreds). The potential for variation in endotoxin potency is an inherent limitation of this model, and accurate reporting of LPS dosage is critical for enabling true comparison between studies.

Saline solution (0.9% sodium chloride) is widely used as a control in studies assessing the effects of intra-articular medications due to its isotonicity and expected minimal biological reactivity. However, there is some evidence that intra-articular saline can itself produce beneficial effects [47]. Moreover, when unbuffered, it can induce nociceptive signalling and hyperalgesia in joint tissues, which could confound interpretations of LPS-induced inflammation [48]. Therefore, it could be argued that control injections using saline should not be performed and that simply the act of needle penetration should be used instead. However, in this study the authors felt that a saline control is appropriate given the LPS is diluted using saline, and the magnitude of any saline-induced inflammatory effect is negligible when compared to LPS. Further research is required to better understand the effects of intra-articular saline and its role as an intra-articular control.

## 5. Conclusions

In conclusion, the findings of this study confirm that this LPS model produces a consistent and reliable synovitis at the 0.25 ng and 0.125 ng dose. However, whilst the 0.125 ng LPS dose did produce comparable increases in TP, WBC, PGE_2_ and MMP activity, there was no statistically significant change from baseline lameness values. It is not possible to conclude that the 0.125 ng dose does not cause lameness, but it is clear that the lameness is less severe and much less consistent. These findings support that the model is valid at both doses and that using lameness as an output measure is not essential. The 0.125 ng dose would be best suited to a bilateral LPS model where lameness is not measurable given the inherent bilateral nature of the study design. Further work is indicated to enhance the clinical relevance of the model to induce synovial biomarker increases without an overzealous inflammatory response, resulting in markedly increased WBC and TP levels that do not accurately reflect clinical disease. To this end, the authors believe that a further reduction in the LPS dose might be possible, or further consideration could be given to the use of repeated low-dose LPS or slow-release mechanisms to better mimic clinical disease. The LPS model provides a platform for the study of inflammatory mechanisms and assessment of the efficacy of anti-inflammatory agents; however, it does not fully replicate the progressive nature of cartilage deterioration, subchondral bone remodelling, and sustained nociceptive states that characterise the natural course of osteoarthritis. A more comprehensive appraisal of a therapeutic’s potential may be achieved by combining the LPS model with complementary surgical models of joint pathology, thereby concurrently reproducing chronic degenerative changes and superimposed acute inflammatory flares. This single-LPS-induction model in its current form is best suited to the study of therapeutics during the acute phase of inflammation. The ultimate aim should be to develop and utilise animal models of disease that are subclinical (do not induce pain), yet accurately reflect clinically relevant aspects of the disease and its in vivo complexities.

## Figures and Tables

**Table 1:** Summary of WBC and TP at peak values (PIH 8) in three different breed populations using a 0.25 ng dose of LPS derived from previously published literature.

Peak Values (PIH 8)	Breed	WBC (×10^9^ cells/L)	TP (g/L)
This Study	TB	116.1 ± 57.6	52.2 ± 11.3
(Van de Water et al., 2021) [8]	SB	126.6 ± 21.8	51.5 ± 6.7
(Kearney et al., 2023) [24]	Mixed	102.8 ± 39.6	56.3 ± 16.12

TB, Thoroughbred; SB, Standardbred.

## Data Availability

The datasets used and/or analysed during the current study are available from the corresponding author on reasonable request.
